# A tale of two neglected systems—structure and function of the thin- and thick-walled sieve tubes in monocotyledonous leaves

**DOI:** 10.3389/fpls.2013.00297

**Published:** 2013-08-06

**Authors:** C. E. J. Botha

**Affiliations:** Department of Botany, Developmental and Applied Plant Anatomy, Rhodes UniversityGrahamstown, South Africa

**Keywords:** early and late metaphloem, thick-walled and thin-walled sieve tubes, monocotyledon, vascular bundle structure

## Abstract

There is a large body of information relating to the ontogeny, development and the vasculature of eudicotyledonous leaves. However, there is less information available concerning the vascular anatomy of monocotyledonous leaves. This is surprising, given that there are two uniquely different phloem systems present in large groups such as grasses and sedges. Monocotyledonous leaves contain marginal, large, intermediate, and small longitudinal veins that are interconnected by numerous transverse veins. The longitudinal veins contain two metaphloem sieve tube types, which, based upon their ontogeny and position within the phloem, are termed early (thin-walled) and late (thick-walled) sieve tubes. Early metaphloem comprises sieve tubes, companion cells and vascular parenchyma (VP) cells, whilst the late metaphloem, contains thick-walled sieve tubes (TSTs) that lack companion cells. TSTs are generally adjacent to, or no more than one cell removed from the metaxylem. Unlike thin-walled sieve tube (ST) -companion cell complexes, TSTs are connected to parenchyma by pore-plasmodesma units and are generally symplasmically isolated from the STs. This paper addresses key structural and functional differences between thin- and thick-walled sieve tubes and explores the unique advantages of alternate transport strategies that this 5–7 million years old dual system may offer. It would seem that these two systems may enhance, add to, or play a significant role in increasing the efficiency of solute retrieval as well as of assimilate transfer.

## Organization of the vein network in monocotyledonous leaves

### Longitudinal vein characteristics

**Figure 1:**
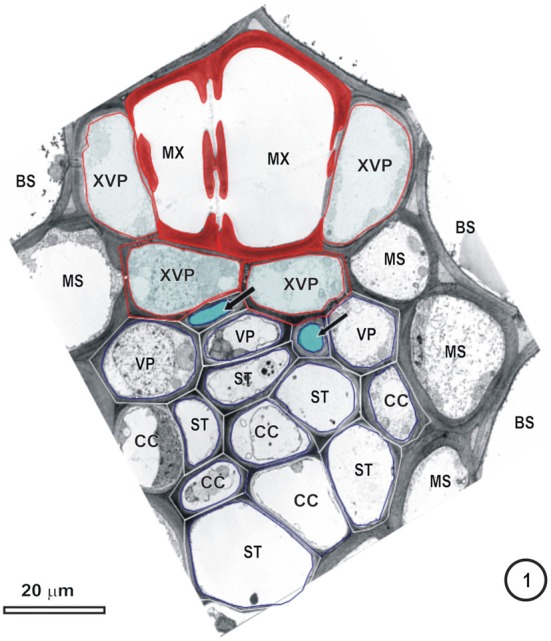
**Transmission electron micrograph of a large intermediate vascular bundle in the rice leaf blade surrounded by a parenchymatous bundle sheath (BS).** The mestome sheath (MS) is only associated with the phloem pole of this vascular bundle. Metaxylem vessels (MX, red cell walls) are associated with two xylem vascular parenchyma cells (XVP, walls outlined in red and with a pale blue fill). Two late-formed, thick-walled metaphloem sieve tubes (unlabeled arrows and blue fill) abut XVPs. The early metaphloem (outlined in white) contains thin-walled sieve tubes (ST) associated with companion cells (CC) and several parenchyma cells (VP).

**Figure 2:**
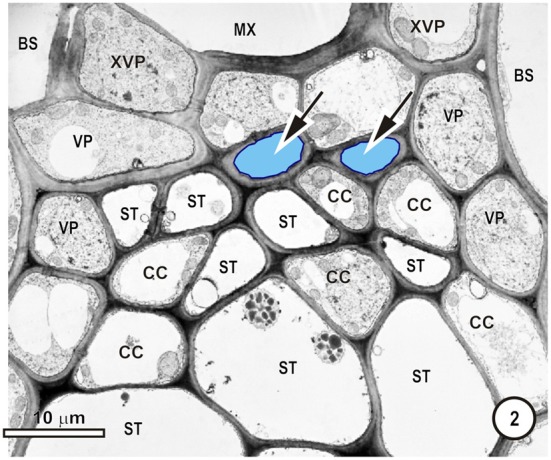
**Detail of the phloem within a large intermediate vascular bundle in the rice leaf blade.** Two thick-walled (TSTs, arrows, blue outline, pale blue fill) and numerous thin-walled (STs) sieve tubes are visible. All STs are associated with companion cells (CC, not all are labeled) and surrounding the ST-CC's are peripherally-located vascular parenchyma (VP), external to which is the bundle sheath (BS). Note that the lower ST are larger than the upper ST.

The vascular bundles in leaves and stems in monocots are described as closed. No secondary vascular differentiation can occur and veins are always collateral. In leaves, longitudinal veins are classified as large, intermediate and small on the basis of the presence or absence of large metaxylem vessels (MX) as well as the presence or absence of protoxylem elements (for a detailed discussion see (Botha et al., 1982) and literature cited). Longitudinal leaf blade bundles tend to intergrade with one another, inasmuch that small intermediate veins may for example, resemble small veins closer to leaf tips (Dannenhoffer et al., 1990; Dannenhoffer and Evert, 1994). The first-formed phloem (protophloem) is generally short-lived and is replaced by metaphloem. The metaphloem contains two distinct functional sieve tube types—described as early metaphloem, which contains thin-walled sieve tubes (ST) which are associated with companion cells and vascular parenchyma (VP) cells, and late metaphloem, which consists of a few thick-walled sieve tubes (TST) that lack companion cell associations. TSTs differentiate in close proximity or adjacent to MXs and connect via pore plasmodesma units (PPUs), to VP cells adjacent to the MX. Two or more TSTs, may be present per vascular bundle (Figures 1, 2 and Dannenhoffer et al., 1990). With the exception of *Commelina* (van Bel et al., 1988) and the Cyperaceae that have been investigated (Botha et al., 2005), no other literature in which the presence of both STs and TSTs in other monocot groups is noted.

Given this unique, two tier sieve-tube arrangement, it is surprising that relatively little information about the two types of monocot sieve tube is discussed in any detail (see Wilson, 1922; Kuo and O'Brien, 1974; Miyake and Maeda, 1976; Cartwright et al., 1977; Evert et al., 1978, 1996; Eleftheriou, 1981, 1990; Evert, 1981; Colbert and Evert, 1982; Evert and Mierzwa, 1989; Dannenhoffer et al., 1990; Botha, 1992; Botha and van Bel, 1992; Bosabalidis et al., 1994; Trivett and Evert, 1998; Nelson and van Bel, 1998). Unfortunately, the presence or distinction between STs and TSTs are sometimes overlooked and if these are recognized, they may be misinterpreted (see Chonan et al., 1981, 1991; Miyake et al., 1982 and review by van Bel, 2003, for example). Wilson (1922) first reported that the phloem in grasses contained lignified sieve elements, as did Lush (1976) for *Lolium tremulentum*; Cartwright et al. (1977) for *Triticum aestivum*; Kuo and O'Brien (1974; Kuo, 1993) for wheat, Eleftheriou (1981) for *Aegilops comosa* (goat grass); Colbert and Evert (1982), for *Saccharum officinarum* and Dannenhoffer et al. (1990) for *Hordeum vulgare*. However, subsequent studies in the same species do not support lignification of the TST walls in these species (see Walsh, 1974; Walsh and Evert, 1975; Evert et al., 1977, 1978, 1996; Heyser et al., 1978; Evert, 1981, 1982; Colbert and Evert, 1982; Fritz et al., 1983; Evert and Mierzwa, 1989; Dannenhoffer et al., 1990; Botha, 1992, 2005; Botha and van Bel, 1992; Evert and Russin, 1993; Matsiliza and Botha, 2002; Botha and Matsiliza, 2004; Scarpella and Meijer, 2004).

Phloem ontogeny has been described in detail by Esau (1976). and by Evert (2007). Whilst it is clear that the ST-CC complex in angiosperms is derived from the same mother cell, frustratingly, no information exists on the ontogeny of the TST, other than reports stating that it is the last differentiated, phloem tissue and is thus referred to as late metaphloem. STs are symplasmically interconnected with CCs via PPUs and only one study Evert et al. (1978) report that connections between TST and CC occur rarely, thus apart from this study, no other evidence from any of the frequency studies exists which supports interconnection of CCs with TSTs or of STs with TSTs—thus, STs are effectively symplasmically isolated from TSTs.

Turgeon and Oparka (1999) described the CC as a “traffic control center” and suggested that these cells may take over the majority of the function from the enucleate SE. van Bel and Knoblauch (2000) described the relationship between CC and SE as that of a “a hyperactive nurse and comatose patient.” Unfortunately, both reviews focus only on SE-CC relationships, not on those between TSTs and their associated VPs.

The earliest record for the presence of TSTs appears in SEM images from fossil leaf fragments of a C_4_ grass apparently that is approximately 5–7 million years old (Thomasson et al., 1986). However, there remains no clear reason or answer to why TST actually evolved or for that matter, why they are still present in monocots and importantly, in two of the largest and most wide-spread groups—the Gramineae and Cyperaceae. This observation demands answers to the following questions: Are both sieve tube types involved in accumulation and long-distance transport and is the relationship between the TST and their VP the same as that between ST and CC? This paper seeks to address these questions and highlights the need for continued in depth research into the neglected sieve tubes in monocots.

### Functional relationships within vascular bundles; longitudinal vascular bundles

**Figure 3:**
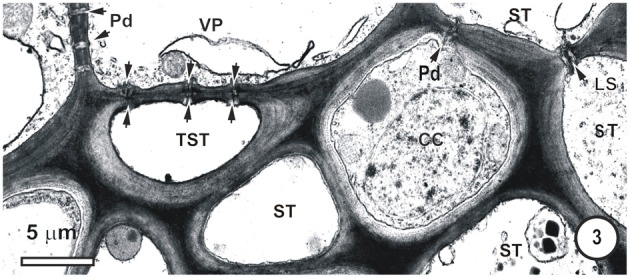
**Detail from a small-intermediate bundle in the rice leaf blade.** Pore-plasmodesma units (paired arrowheads, left) connect TST at left, to the VP cell above. The ST (upper right) is connected via PPUs to a companion cell (CC) via PPU (paired arrowheads, upper right) and the two ST (right) are connected via a lateral sieve area (LS). Plasmodesmata (Pd, left) interface VP cells. No connections between thick-and thin-walled sieve tubes are visible in this TEM image.

**Figure 4:**
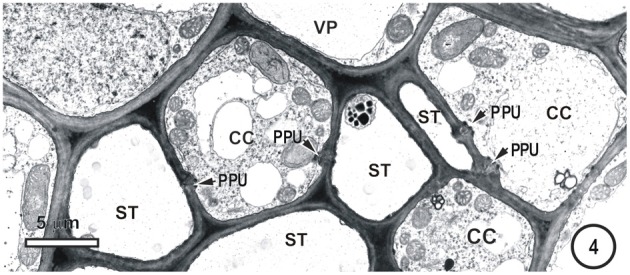
**Detail showing a field containing several thin-walled sieve tubes, associated vascular parenchyma (VP) and companion cells (CC).** Thin-walled sieve tubes (ST) are connected to CC by pore-plasmodesmata units (PPU).

**Figure 5:**
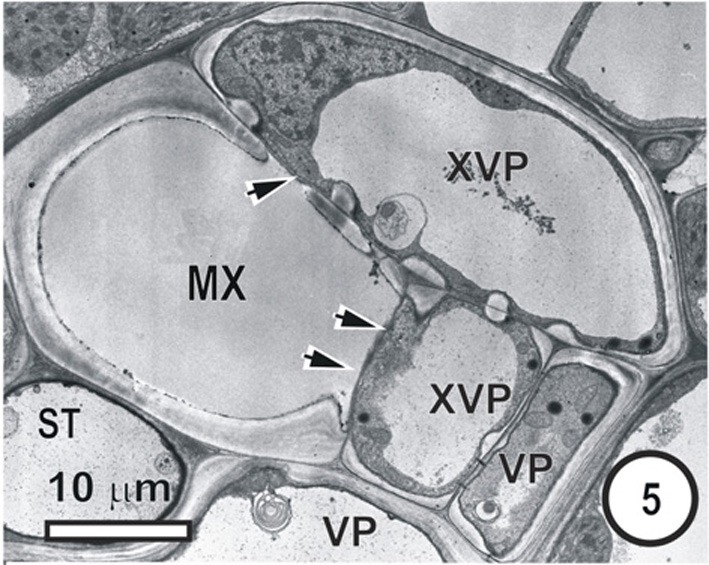
**Aspects of anatomy and cell associations in a small transverse vein in the leaf blade of *Saccharum officinarum* (NCO 376).** This vein contains one metaxylem vessel (MX) coupled via prominent half-bordered pit pairs (arrowheads) to xylem vascular parenchyma cells (XVP), which, in turn, are symplasmically associated with several vascular parenchyma cells (VP). A solitary sieve tube is next to the MX. Judging by the thin wall it is in all probability an ST.

**Figure 6:**
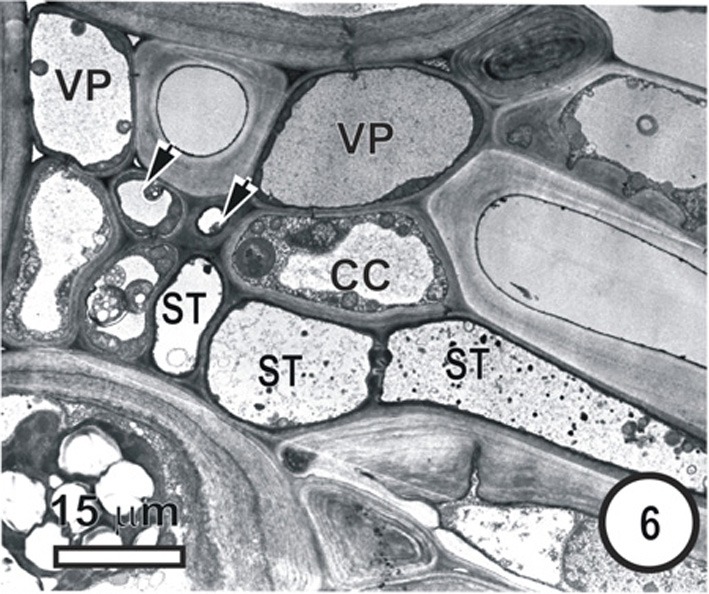
**Aspects of anatomy and cell associations in a transverse section through part of an intermediate vascular bundle (left) from a mature *Panicum maximum*leaf blade, cut at the level of an emerging transverse vein sieve tube (ST, to right).** Based on its position, this is also thin-walled. Two TST (arrowheads) and several ST are visible in the longitudinal bundle.

**Figure 7:**
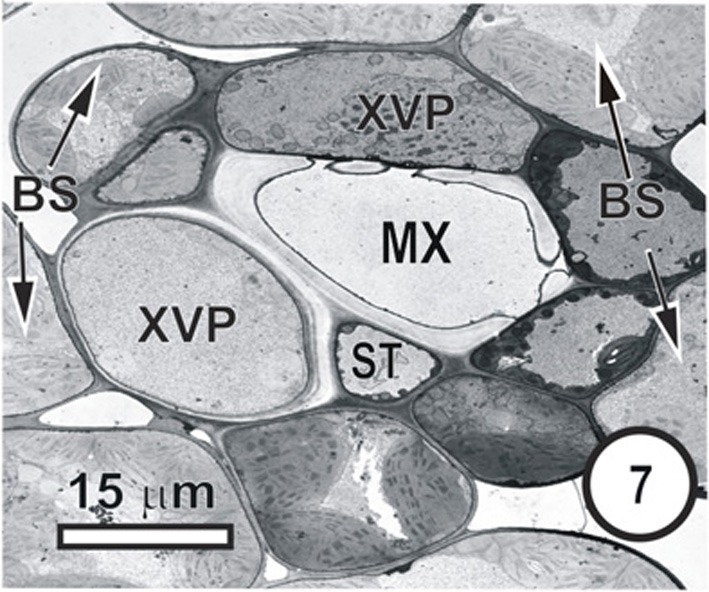
**Aspects of anatomy and cell associations in a transverse vein in a *Bromus unioloides* leaf.** A single sieve tube is adjacent to a solitary xylem vessel, which is in contact with (XVP) that are in contact with the single vessel (XV). As is common in many grasses, the bundle sheath is incomplete, and the BS is interspersed with prominent intercellular spaces.

**Figure 8:**
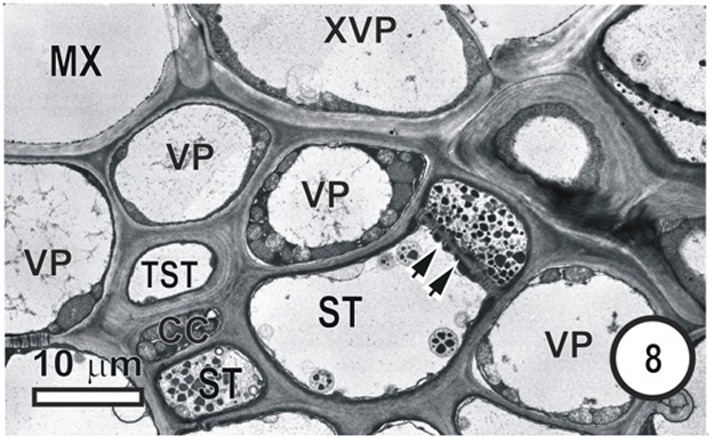
**Aspects of anatomy and cell associations in a small intermediate vein in *Eragrostis plana*.** Part of two metaxylem vessels (MX, extreme left top and right top) are separated from the phloem by XVP. This vein contains a single TST and several ST. Lateral sieve area pores (paired arrowheads) connect the two central STs.

**Figure 9:**
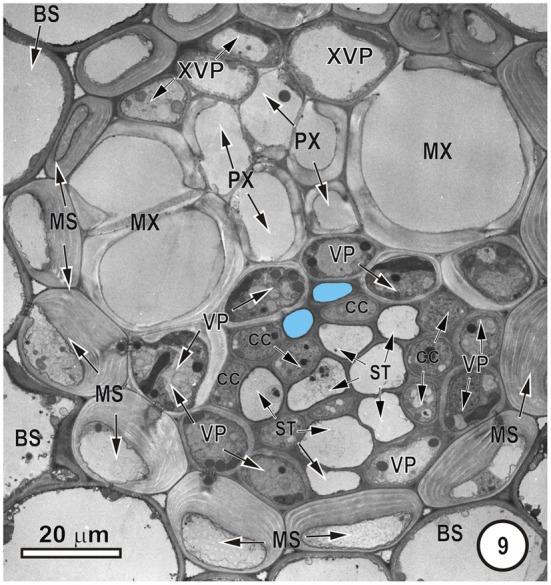
**Intermediate vascular bundle in the leaf blade of *Mariscus congestus* (Cyperaceae)**. The vascular tissue is surrounded by two sheaths—an outer parenchymatous bundle sheath (BS) and an inner lignified mestome sheath (MS). Note extreme thickening of inner tangential walls of MS cells. Only two thick-walled sieve tubes (pale blue fill), that are separated from the MX by a single row of XVP. MX are associated with smaller protoxylem vessels (PX). STs are associated with companion cells (CC) and are bordered by VP.

Figures 1–9 depict the cell arrangement in the various vein orders within the Poaceae and Cyperaceae leaves with particular focus on the distribution of their ST-CCs and TST-VPs. Vascular bundles are surrounded by a bundle sheath (BS), and large as well as intermediate bundles may contain a mestome sheath (MS, Figure 1). MX (Figure 1) are always associated with a few VP cells that abut the vessels and which interconnect to the metaxylem through hydrolyzed half bordered pit-pairs. These xylem parenchyma cells serve as a conduit between the xylem and parenchymatous elements including sieve tubes and because of their potentially important role in transport, (see Evert et al. (1978); Fritz et al. (1983); Botha et al. (2005, 2008) are termed xylem vascular parenchyma (XVP), to distinguish them from other VP. Typically, numerous plasmodesmata interface these xylem-associated XVPs to the TSTs. The large intermediate bundles (Figure 2) in rice leaves usually contain two TSTs (Figure 2). In most grasses and sedges, TSTs are connected to XVPs (Figures 2, 3) by PPU-like connections and TSTs are interconnected via lateral sieve area pores. No connections have been seen between STs and TSTs. Symplasmic isolation between STs and TSTs has been reported in *Zea mays* (Evert et al., 1978), in several grass species (see Botha, 1992; Botha and van Bel, 1992; Botha and Cross, 1997) and in rice where STs show no evidence of 5,6-CF uptake (Figures 14–16).

Studies of plasmodesmal patterns in grass leaves (see Evert literature, Botha and van Bel, 1992; Botha and Cross, 1997) suggest that there are apparently no direct connections between the TSTs and STs, which implies that there may be two independent transport pathways within the same vascular bundle. However, this does not rule out the possibility that very low frequencies of small sieve pore areas could well exist and could possibly connect the two sieve tube types infrequently that could be missed even during careful examination of thin sections at the TEM level. The question that remains unanswered to this point, is therefore, is it possible to demonstrate unequivocally, that there are no functional connections between STs and TSTs—either directly between the two sieve tube types, or indirectly via their concomitant VPs?

### Functional relationships within vascular bundles; transverse veins

**Figure 10:**
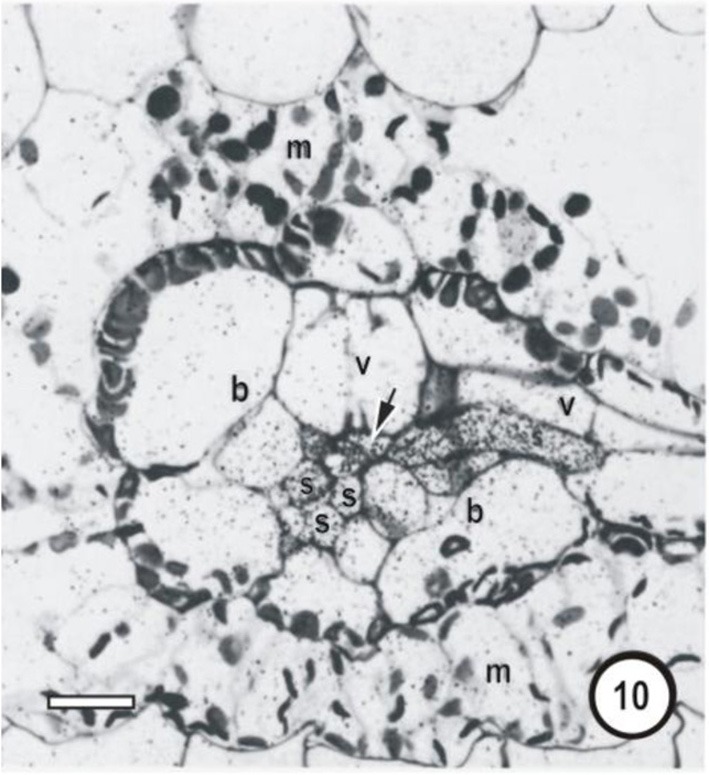
**Microautoradiographs of small vascular bundles in a *Zea mays* leaf and portions of transverse veins after 5 min feeding with ^14^*CO*_2_, followed by a 10-min ^12^*CO*_2_ chase showing accumulation of the ^14^C label in thick- and thin-walled sieve tubes.** Label has accumulated in phloem cells as well as being transferred to an emergent thick-walled transverse vein sieve tube to the right, which enters the small bundle at the level of the thick-walled sieve tube.

**Figure 11:**
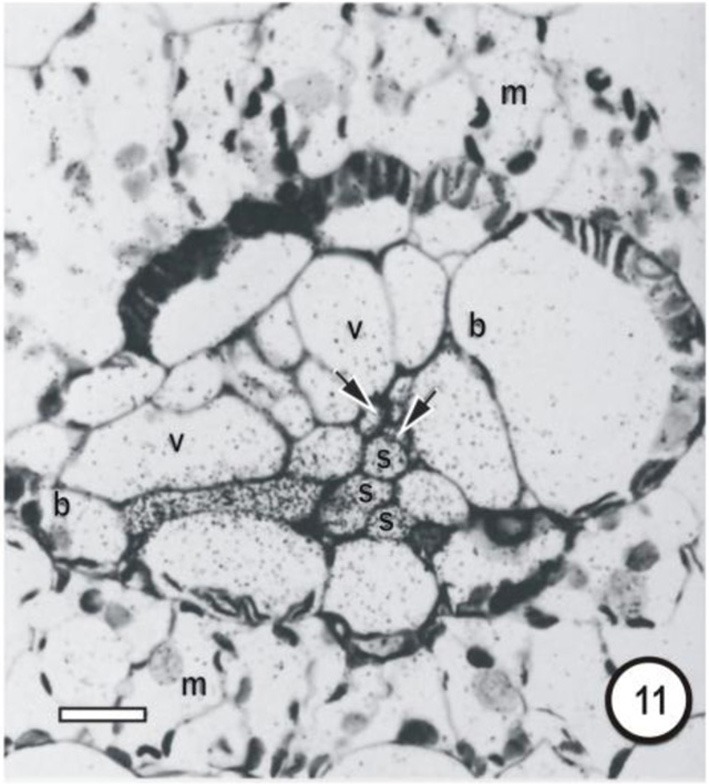
**Microautoradiographs of small vascular bundles in a *Zea mays* leaf and portions of transverse veins after 5 min feeding with ^14^CO_2_, followed by a 10-min ^12^CO_2_ chase showing accumulation of the ^14^C label in thick- and thin-walled sieve tubes**. The sieve tube of transverse vein enters the bundle at the level of the thin-walled sieve tubes (STs). Label has accumulated in STs and has been exported to a transverse vein ST. b, bundle-sheath cell; m, mesophyll cell; s, thin-walled sieve tube; v, vessel; unlabeled arrows point to thick-walled sieve tubes; bars = 16μm. Adapted and used with permission of the publishers (Springer; License No 2795360987331) from Fritz et al. (1983). Labeling as in original paper and for details, see Fritz et al. (1983).

**Figure 12:**
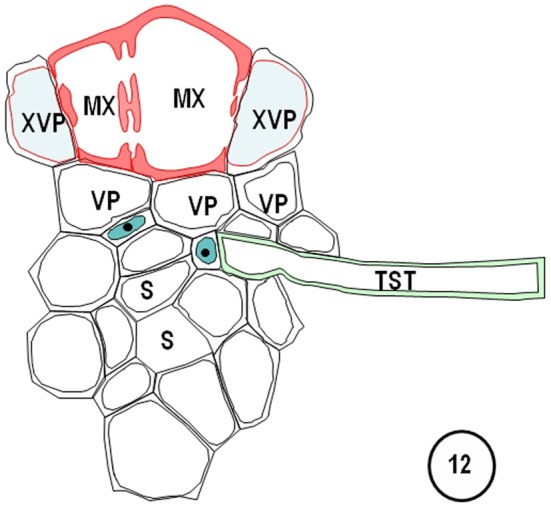
**Diagram redrawn and interpreted from Figure 10, showing a thick-walled sieve tube (blue fill, solid dot) connection to a transverse vein TST (left).** The emergences of a transverse vein ST from its longitudinal vein connection. XVPs (with half-bordered pit-pairs) about the xylem (MX) and vascular parenchyma (VP) are in association with the TSTs.

**Figure 13:**
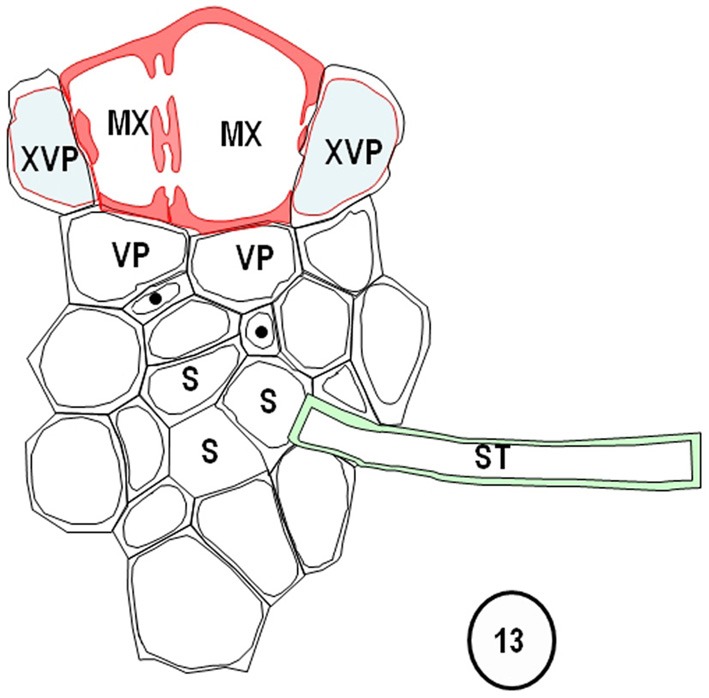
**Diagrams redrawn and interpreted from Figure 11, showing a thin-walled sieve tube (S) connection to a transverse vein ST.** The emergences of a transverse vein ST from its longitudinal vein connection. XVPs (with half-bordered pit-pairs) about the xylem (MX) and vascular parenchyma (VP) are in association with the TSTs.

**Figure 14:**
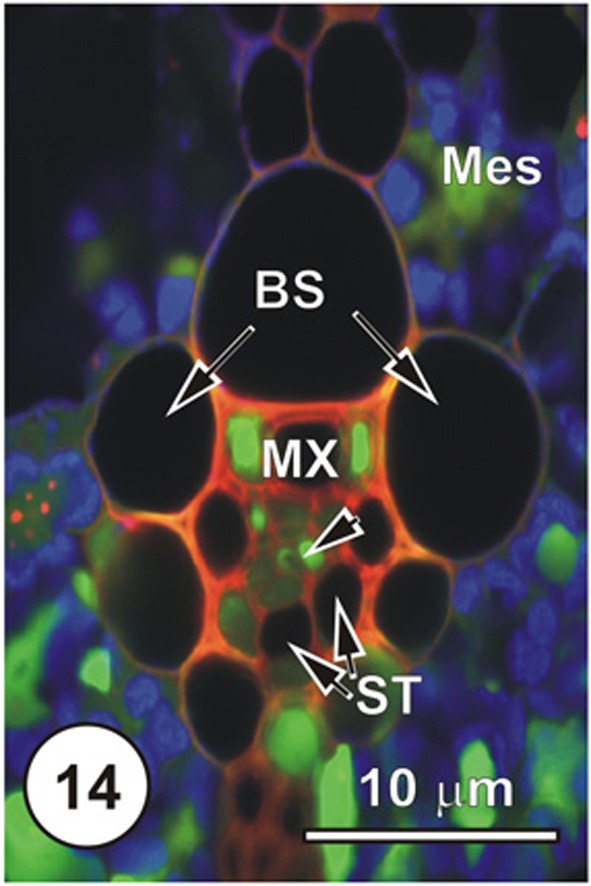
**LSCM imaged transections through a small (Figure 14), an intermediate (Figure 15) and a large (Figure 16) longitudinal bundles, showing distribution of 5,6-CF, after uptake of 5,6-CFDA (green fluorescence) co-transported with propidium iodide (red fluorescence; PI stains lignin and cellulosic walls) after 45 min uptake and 6 cm above the cut end of a rice leaf blade [full methodology in Botha et al. (2008)].** Chloroplast- containing mesophyll cells (Mes) have been false colored to blue to prevent interference with propidium iodide or the 5,6-CF dyes. Serial sections were cut directly into silicone oil and sections were covered with coverslips prior to examination using a Leica SP2 LCSM. Propidium iodide selectively stained lignified walls (red fluorescence). Small vein, showing that 5,6-CF had been offloaded into xylem parenchyma, either side of the solitary metaxylem vessel (MX) Note that the two thin-walled sieve tubes (ST, abaxial) contain no evidence of the probe, but thick-walled sieve tubes (dart below the metaxylem vessels). There is no evidence that 5,6-CF has offloaded to the bundle sheath (BS), but it is evident as well as in mesophyll (Mes), indicating symplasmic transport outwards into the mesophyll.

**Figure 15:**
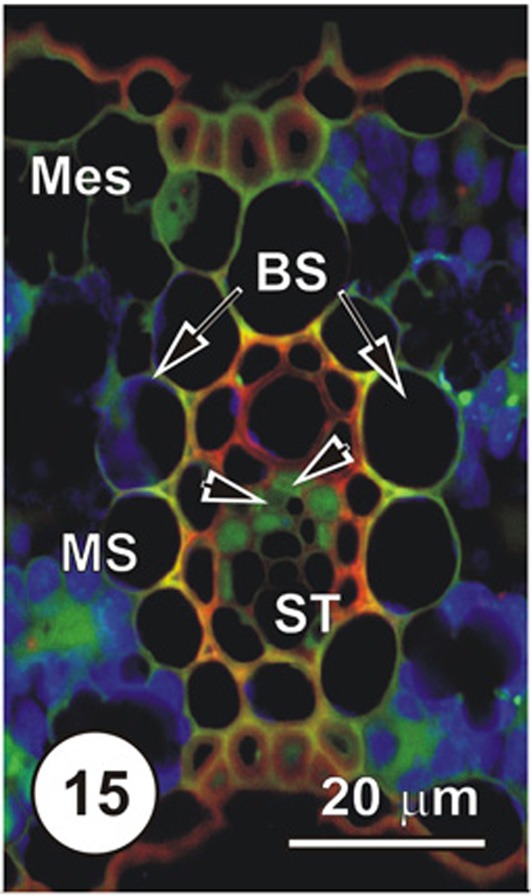
**LSCM imaged transections through a small (Figure 14), an intermediate (Figure 15) and a large (Figure 16) longitudinal bundles, showing distribution of 5,6-CF, after uptake of 5,6-CFDA (green fluorescence) co-transported with propidium iodide (red fluorescence; PI stains lignin and cellulosic walls) after 45 min uptake and 6 cm above the cut end of a rice leaf blade [full methodology in Botha et al. (2008)].** Again, there is no evidence of 5,6-CF in ST, but TST and concomitant VP contain the green fluorescence (arrowheads) 5,6-CF is also present in mesophyll cells (Mes).

**Figure 16:**
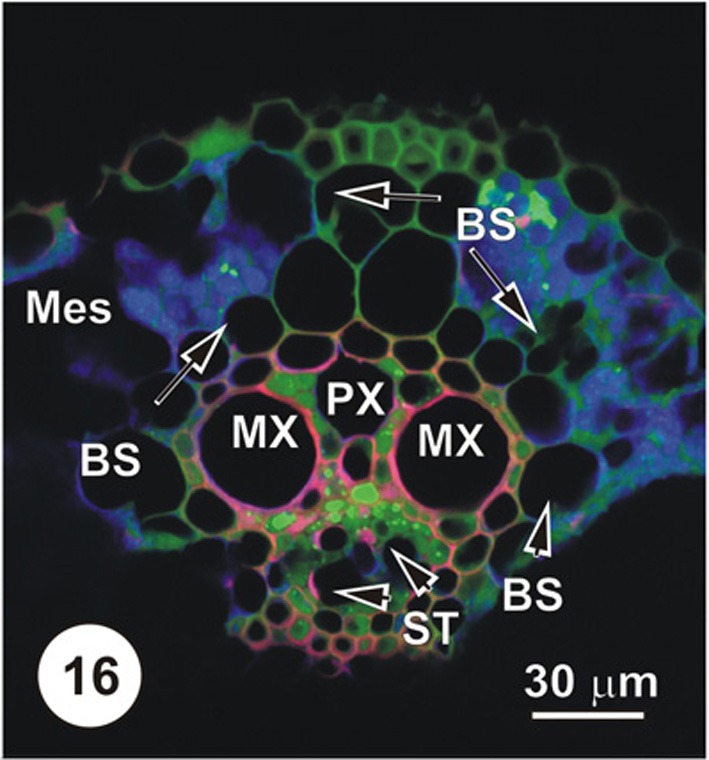
**LSCM imaged transections through a small (Figure 14), an intermediate (Figure 15) and a large (Figure 16) longitudinal bundles, showing distribution of 5,6-CF, after uptake of 5,6-CFDA (green fluorescence) co-transported with propidium iodide (red fluorescence; PI stains lignin and cellulosic walls) after 45 min uptake and 6 cm above the cut end of a rice leaf blade [full methodology in Botha et al. (2008)].** Vascular parenchyma associated with the protoxylem (PX) contains 5,6-CF, as do cells immediately below the MX, where VP and TST contain 5,6-CF (green fluorescence). The ST and below these, large diameter early ST (arrowheads) contain no 5,6-CF. Note that some 5,6-CF is present in the hypodermal fibers associated with this vascular bundle. As is common in grasses, vascular bundles are surrounded by a bundle sheath (BS).

**Figure 17:**
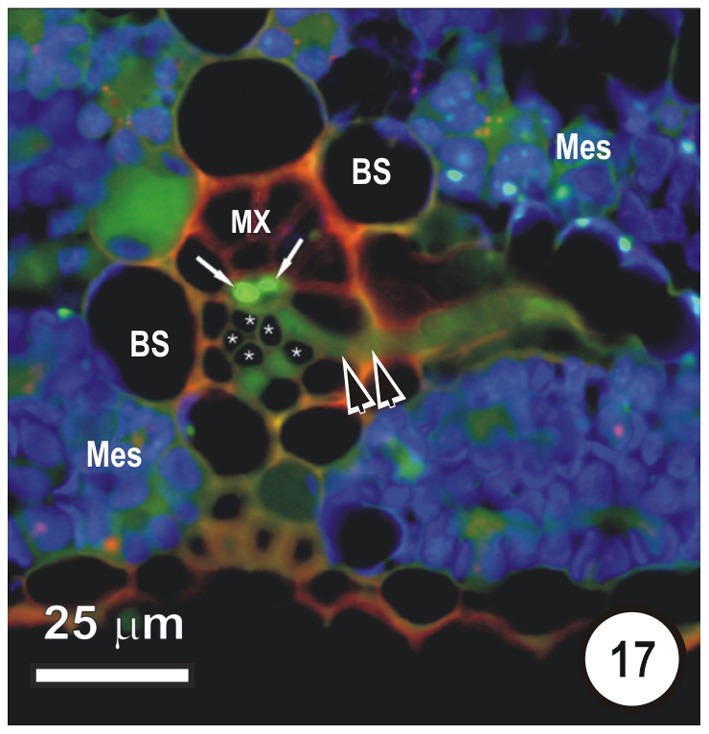
**Transection of a small intermediate transverse vein in a rice leaf, showing the distribution of 5,6-CF after uptake of 5,6-CFDA in the transpiration stream.** 5,6-CFDA was co-transported with a weak solution of propidium iodide (red fluorescence) via the xylem for 30 min. An emergent thick-walled sieve tube (paired darts), together with two longitudinal TST (arrows), contains 5,6-CF (green fluorescence). Thin walled sieve tubes (asterisks) do not contain 5,6-CF. Note that some 5,6-CF has transported symplasmically, through the bundle sheath (B) to the mesophyll (Mes). Preparation as per Figures 14–16.

Transverse veins emerge from longitudinal veins and interconnect all three longitudinal bundle orders. They may be partially or completely surrounded by a parenchymatous bundle sheath (see Figures 1–9, Figures 14–17). In grasses and sedges, transverse veins will usually contain a single xylem vessel, accompanied by a single sieve tube and they share a common interface to one or more VP cells. The sieve tubes may be either thin- or thick-walled and the type of the location and sieve tube type of the emergent sieve tube within the longitudinal vein; STs always connect to a ST and TSTs always connect to a TST (see Figures 10, 11; Fritz et al., 1983). Figures 12 and 13 are diagrammatic representations of the connections of longitudinal vein TST to transverse vein TST, and of longitudinal vein ST to transverse vein ST, re-interpreted from Figures 10 and 11.

Transverse veins form an extensive system (up to 16% of total vein length in the barley leaf (Dannenhoffer et al., 1990). Their extensive interconnections with longitudinal veins together with the capacity to transport ^14^C–sucrose in either STs or TSTs, suggest that both could act as distributaries or tributaries of the longitudinal veins. However, Fritz et al. (1983) argue that it is doubtful that transverse veins serve any storage function as previously suggested by Lush (1976), but could be involved in sucrose buffering and shunting of assimilate via the interconnecting transverse veins thereby maintaining balance, which would prevent a build up of sucrose in longitudinal vein STs or TSTs.

## Experimental studies on the roles of STs and TSTs

### ^14^C uptake and transport studies

There is direct evidence that ^14^C-assimilates are almost exclusively translocated through the STs and not the TSTs. Cartwright et al. (1977) suggested that the “lignified” sieve elements (TSTs) were not involved in storage, or in translocation, unless the process was very rapid or alternatively, very slow. In later experiments, Fritz et al. (1983) fed ^14^C–sucrose directly to cut leaves of *Z. mays*. They showed that ^14^C–Sucrose was taken up by the xylem transpiration stream and that direct transfer took place to neighboring XVPs, through the half bordered pit-pairs between the MX and the XVP. Furthermore, the first sieve tubes to accumulate ^14^C–sucrose were the TSTs contiguous to the XVPs. With increasing time, a greater proportion of the TSs took up the ^14^C label. Exposing *Z. mays* leaves to ^14^*CO*_2_, showed that all phloem cells, regardless of position, took up label and that with time, a greater proportion of ^14^C was accumulated within STs. These data collectively support a long-distance function for STs and for higher levels translocated sugars (see discussion in Colbert and Evert, 1982; Russell and Evert, 1985) in the ST.

### What do aphid feeding experiments tell us?

Matsiliza and Botha (2002) reported that the common grass aphid *Sitobion yakini* fed preferentially from STs and that 84% of stylets and 92% of the stylet tracks, terminated in, or at STs. Of interest, 55% of the aphid probes terminated in small veins (Matsiliza, 2003). In a subsequent study, Botha and Matsiliza (2004) reported that the Russian wheat aphid, *Diuraphis noxia*, like *S. yakini*, showed no preference for the TSTs in wheat leaves either. Clearly, preferential feeding on ST is difficult to explain other than that the (a) wall structure of the TST is difficult to breach, or (b) the contents of the TST are unpalatable to aphids. However, since all feeding studies show that aphids will penetrate lignified xylem vessels and tracheids from time to time to drink watery solutes, implies that wall thickness itself, cannot be the reason for the unattractiveness of the TSTs. Why then are TSTs unattractive to aphids?

Given that the relative number of TSTs decreases with decreasing vein size, to the point where there may only be a solitary TST in the small longitudinal veins (Colbert and Evert, 1982; Russell and Evert, 1985) as well as the direct evidence that 14C-assimilates are almost exclusively translocated through the STs and not the TSTs (Cartwright et al., 1977) makes them attractive to aphids.

Logically, it seems that TSTs may simply not be attractive enough to aphids, given that aphids may follow a sucrose concentration “trail” to functional phloem which would be provided in the apoplasmically-loaded STs and their higher, more attractive sucrose content. Thus, preferential feeding on the STs suggests that carbohydrate levels, at least in TST are unattractive. Recent work by Hewer et al. (2010) convincingly demonstrated that when aphids were fed artificial diets, their general preference was for the artificial diet that most closely matched that of their host plants. In addition, Hewer et al. (2010) demonstrated that the aphids were attracted solely by carbohydrate abundance as well as a neutral to slightly alkaline pH. The unattractiveness of the TSTs as feeding sites might therefore also be for the same reason.

### Plasmolytic studies

Evert et al. (1978) combined plasmolytic examination and TEM to determine osmotic potentials of phloem cell sap of mature *Z. mays* leaves and they reported that the osmotic potentials of CC-ST complexes of leaves sampled from lighted plants, plasmolysed in a 600 mM sucrose solution (−0.18 MPa or −1.8 bar) and the surrounding VPs plasmolysed in a at about 400 mM sucrose solution (0.11 MPa; or −1.1 bar). In the veins from 48 h dark pre-treatment, ST-CCs as well as associated VPs plasmolysed in a 200 mM (0.06 MPa or −0.6 bar), sucrose solution but, surprisingly, the TSTs and their associated VPs did not, which is added support for the symplasmic isolation of TSTs from STs.

### What do sucrose transporters inform us of?

**Figure 18:**
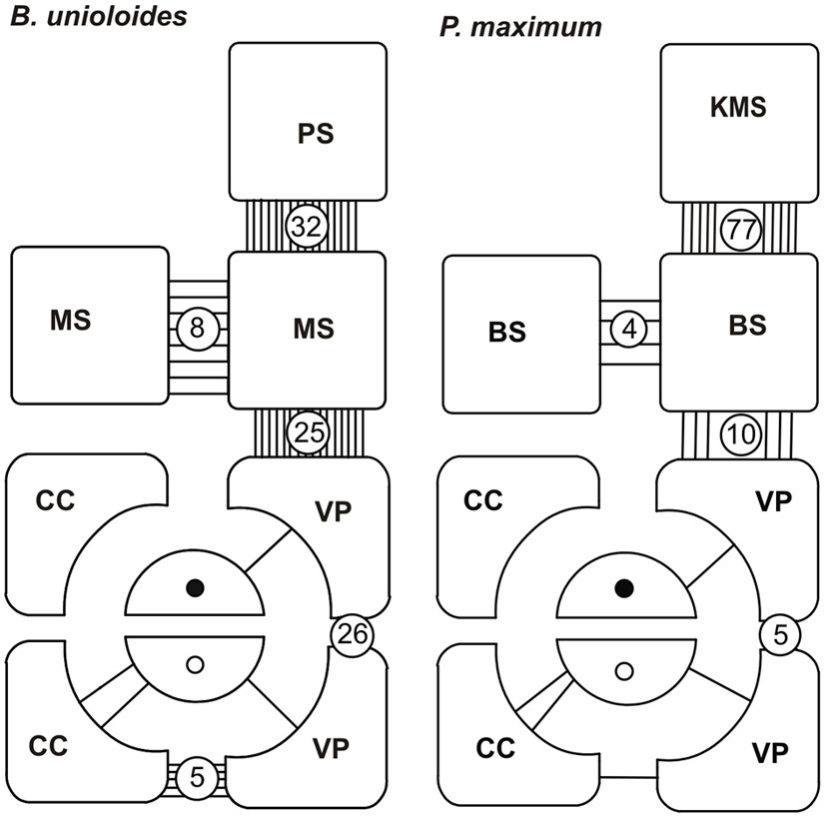
**Plasmodesmograms showing the distribution of plasmodesmata, expressed as percent plasmodesmata/μm cell-wall interface for the C_3_ grass, *Bromus unioloides*and the C_4_ grass *Panicum maximum* expressed as percent plasmodesmata/μm vein.** *B. unioloides* has a double sheath—an outer parenchyma sheath (PS) and an inner thick-walled mestome sheath (MS). In *Panicum maximum* the Kranz mesophyll sheath (KMS) surrounds the inner bundle sheath (BS). Note that thick-walled sieve tubes (TSTs, solid dots) do not have connections to the ST-CC (open circles) complex. STs and TSTs have low frequency connections to vascular parenchyma cells (VP). Redrawn from Botha and van Bel (1992).

It is well known that the majority of grasses utilize an apoplasmic phloem loading mechanism, because of the almost complete isolation of the ST-CC complexes from the associated VP (Figure 18). According to Braun and Slewinski (2009) uptake of sugar requires an apoplasmic step, whereby the sugar is exported from the VP, into the free space and almost certainly sucrose transporters are implicated in uptake into the ST-CC complex. In recent years, SUT1 (a sucrose carrier) has been shown to be expressed in various phloem cells of a number of plants (see Figure 2 in Braun and Slewinski, 2009, for a phylogenetic tree of grass and selected dicot SUT's). Sucrose transporters have also been identified amongst the monocotyledons, including maize (Aoki et al., 1999), wheat (Aoki et al., 2002), barley (Weshke et al., 2000), and more recently, rice (OsSUT1, Scofield et al., 2007a,b). Scofield et al. (2007b) proposed that OsSUT1 was involved in phloem loading or retrieval in the flag leaf and sheath vascular bundles of rice plants and that OsSUT1 epitopes labeled by an OsSUT1 promoter::GUS construct were almost entirely associated with the phloem tissue, including companion cells and TSTs and/or associated parenchyma. Braun and Slewinski (2009) suggested that SUTs (SUT1 in particular) facilitate sucrose transport across the phloem cell (presumably CC) plasma membrane. Given that four SUT proteins (SUT1, 3, 4 and 5, Braun and Slewinski, 2009) have been identified for *Z. mays* (Aoki et al., 1999, 2003), so it is highly likely that transfer from the VP to the ST-CC complex involves a sucrose/H^+^ symporter such as the *Zm*SUT1 protein.

### Relating plasmodesmal frequencies and xenobiotic transport studies using 5,6-CFDA

Plasmodesmograms *per se* should not be taken as finite interpretations of frequency, but rather as an indication of the connectivity that exists between cell types and thus they provide useful information that should be tested using symplasmically-transported xenobiotics for example. What is abundantly clear from plasmodesmograms, as well as the examination of a large number of sections at the TEM level, is the lack of plasmodesmal interconnectivity between STs and TSTs. This has been determined for many species and has been reported elsewhere (see Botha and van Bel, 1992 and literature cited) and is illustrated here by the plasmodesmograms (Figure 18) for *Bromus unioloides* and *Panicum maximum*. However, it is important to remember that connections between sieve tubes and their associated parenchyma elements—including companion cells—usually occur as aggregates and that these aggregated fields are not necessarily regularly spaced. Thus, “frequencies” depend very much on the number of sections examined to determine PD frequency. Thus, whilst the frequency of connections between VPs and STs, or XVPs and TSTs, can/may be as low as the 1–2% of the total plasmodesmata counted at the ST-VP interface, is indicative of a high probability of symplasmic isolation from surrounding cells (Figure 18 and Evert et al., 1978; Fritz et al., 1983; Botha, 1992; Botha and van Bel, 1992; Botha and Cross, 1997). Thus, plasmodesmatal frequency studies provide strong (but circumstantial) evidence for (a) isolation of the TST from ST; (b) isolation of the TSTs from CC; (c) Variable PPU frequency between the TSTs and XVPs (d) low frequencies between the VP and CC. Based upon the plasmodesmatal frequency studies it appears then that loading of the TSTs is symplasmic which is in contrast to the ST, where loading will in all probability involve an apoplasmic loading step and must involve membrane transporters (Aoki et al., 1999; Turgeon and Oparka, 1999; Scofield et al., 2007b). However, unless functional transport pathways (or alternatively, lack of transport between particular cell types) can be demonstrated, the information obtained fromplasmodesmatal frequency studies is of limited use. There is general agreement that symplasmic continuity is provided by functional plasmodesmata and that plasmodesmata provide an essential line of communication between living cells. There is a large body of evidence, enhanced in recent years through the careful choice of membrane impermeable fluorescent probes, to test and illuminate transport pathways. This has allowed resulted in refinement of the understanding of intercellular pathways in plants (see Wright and Oparka, 1996 and literature cited). The xenobiotic 5,6-carboxyfluorescein diacetate (5,6-CFDA) is an excellent example of a membrane-permeant, non-fluorescent compound, which, on uptake into living cells, is cleaved of its acetate and the resultant product fluorescent 5,6-CF, is membrane-impermeant (Grignon et al., 1989). Application can be through damaged cells, for example, via “windows” or “flap feeding” cut into the epidermis (Botha, 2005), or it could alternatively be fed directly to the transpiration stream (Botha et al., 2008). Both approaches provide excellent evidence for uptake and transport from cell to cell. In the first instance, post-cleavage transport of fluorescent 5,6-CF from the point of application of 5,6-CFDA into intact mesophyll cells, must take place via plasmodesmata and will therefore illuminate the symplasmic assimilate transport and phloem loading pathways from the mesophyll via the Me -> BS -> VP and dependent on functional plasmodesmata, uptake by the ST-CC complexes. So, low plasmodesmatal frequencies at the VP-CC or VP-ST interfaces, can be tested to see if a functional symplasmic pathway at the VP-> ST-CC exists or not (see Botha, 2005 and literature cited).

In contrast, transport via the transpiration stream results in offloading from the MX, through half-bordered pit-pairs in the common walls between vessels and their associated xylem parenchyma. The 5,6-CFDA is transferred across the half-bordered pit pairs between the MX and the adjacent XVP by diffusion and once in the XVP, 5,6-CFDA is cleaved and any subsequent transport of the membrane-impermeable 5,6-CF, must follow a symplasmic route through PPUs or PD. This application method (Botha et al., 2008) has provided good information on the symplasmic cell connectivity from the XVP to the TST. Irrespective of the approach, and provided functional PD and or PPUs exist, then probes such as 5,6-CF are very useful and will illuminate pathways which terminate in phloem tissue, provided a symplasmic pathway is operative.

It is important to realize though that neither method can rule out diffusion of 5,6-CFDA across several cell-cell boundaries with any absolute degree of certainty and caution is always necessary in interpreting results.

Interpreting flap feeding experiments must take into account the physiological state of the leaf—i.e., whether it is in a source or a sink state. If the rate of photosynthesis and basipetal export from the leaf is rapid, then (depending on functional Pd) symplasmic loading of ST could occur, as assimilates would be moving from the mesophyll down a concentration gradient. Thus, a PD frequency of 1–2% at the VP-CC interface (see Figure 18) may well be adequate to service transport and probes such as 5,6-CF will be taken up by the ST (see Botha and Matsiliza, 2004; Botha, 2005 and literature cited). In contrast, uptake via the transpiration stream will reveal sites at which the probes are offloaded from the xylem vessels and, given sufficient time, the ultimate destination of the probe. Precautions are, however, needed in interpretation and sections taken for transport studies involving water soluble probes should be cut into silicone oil and sections should be taken a reasonable distance from the cut ends of the leaves. If these precautions are observed then there is no reason to expect, nor has any evidence ever been found (see Botha et al., 2008) of uptake-related artifacts. Non-invasive methods yield consistent and highly repeatable results (Figures 14–17) using either wide field fluorescence or laser scanning confocal microscopy (LSCM). In support of this, Botha et al. (2008) found that transport via the xylem transpiration stream in rice leaves was fairly rapid and that within 30–45 min, probes such as propidium iodide and Texas red would co-transport with 5,6-CFDA, that these could be imaged within the same sections, and the ‘dye front’ was usually about 3–5 cm from the point of application. After offloading from the xylem, LSCM and wide-field fluorescence images consistently show that 5,6-CF is trafficked from cell to cell, via plasmodesmata and pore-plasmodesmata. After transport in the xylem, cleaved 5,6-CF never accumulates in STs, and thus provides convincing support for the isolation of STs from TSTs in all grasses examined to date.

### What then, is the function of TSTs?

What then is the role of the TST, other than accumulation of leaked solute? Fritz et al. (1983) provide good argument for lack of involvement of the TST in longitudinal transport. They proposed that TST are involved in rapid transfer of accumulated solute laterally to the ST and that this takes place largely in the transverse veins (see Figures 10, 11, 17). The evidence for this is that contacts were observed between sieve tubes in transverse bundles and both TST and ST of longitudinal bundles. Furthermore, Fritz et al. (1983) state that it is doubtful that transverse veins serve any storage function as previously suggested by Lush (1976) for *Lolium temulentum* (a C_3_ grass) and *Panicum maximum* (a C_4_ grass). From the available evidence, it appears that (a) VP cells (associated with the xylem) are able to retrieve at least sucrose from the vessels and to transfer it to the TSTs; (b) that TSTs are not involved in long-distance transport; (3) that xenobiotics such as 5,6-CF retrieved from the xylem, are not shunted from TSTs to STs (evidence for symplasmic isolation) and (4) that TSTs may be involved in a lateral shunt of retrieved assimilates (and of probes such as cleaved 5,6-CFDA) to adjacent longitudinal veins.

## Concluding remarks

It is strange that so little attention has been directed toward a clearer understanding of short and long-distance transport in Gramineae, given their economic importance. The fact that two sieve tubes with different ontogeny, have existed within monocotyledonous leaf blades for 5–7 million years leads to the conclusion that they are not merely long-forgotten leftovers of some long-defunct transport system, but rather serve different functions.

It is also absolutely clear that generalizations cannot be made, nor can conclusions be drawn that are based solely on the dicotyledonous model of an SE–CC complex. The lack of CC relationships with TSTs (even though they are incorrectly reported to exist in barley; Haupt et al., 2001) precludes either generalization, or direct translation of the SE-CC dogma applied to dicotyledons, to the monocotyledons. The evidence points to two distinct systems, with distinct roles in the monocotyledons studied thus far. Unfortunately, the literature contains a number of perplexing, contradictory reports that do not help when attempting to assign roles to STs or TSTs. For example, Kuo and O'Brien (1974) concluded that the “lignified” sieve elements (assuming that these are TSTs) of the wheat leaf took up more ^14^C–label than did non-lignified ones (assuming that these are STs). In contrast, Cartwright et al. (1977) reported that significantly higher ^14^C activity was associated with “normal” (i.e., thin-walled) sieve elements in wheat leaves than the apparently lignified sieve elements. Whilst it is possible that the experiments reported in these papers were carried out under different conditions, (for example, leaf anatomy/cell maturity will differ with age) contradictions do nothing to help resolve the roles of STs and TSTs.

All of the aphid-based studies of feeding on small grain plants have demonstrated very clearly, that these insects are not attracted to TSTs as a source of food. Instead, aphids overwhelmingly and preferentially probe for, and feed from, STs. Counter arguments are presented that the “thick” walls are somehow impenetrable, hence their lack of “favor” by aphids. This is clearly not so, and all that is necessary is to examine the extensive damage that aphids cause to xylem vessels, in their search for water. Probing for water, causes catastrophic damage to vessels and their associated xylem parenchyma cells (Saheed et al., 2007). Based on this, lignin clearly, does not present a problem and the answer to the lack of interest in TST must lie elsewhere. The preferential penetration of STs therefore suggests that aphids are attracted by the higher concentration of sucrose in STs. As mentioned, rapid shunting of solutes from TSTs (see Evert et al., 1978) implies a lower sucrose concentration in the TSTs, compared with STs. TSTs appear to be involved in recovery and transfer of retrieved solutes via the XVP to TSTs. According to Heyser et al. (1977) and Evert et al. (1978), a transient shunt in the transverse veins is involved in recovery to TSs in adjacent vascular bundles. Transfer to STs thus implies that TSTs have no function in long distance transport, other than in retrieval and recovery of sugars from the xylem.

### In summary

The STs and TSTs appear to serve unique functions.

1. XVPs are able to retrieve sucrose from the xylem apoplasm and to transfer this to TSTs.
2. TSTs are apparently not involved in long-distance transport, but act to shunt retrieved sugars via transverse vein sieve tubes, to large transport vein STs.
3. Given the low Pd frequencies, and the association of SUTs with the phloem is not surprising, as STs may accumulate sucrose and photoassimilates directly from the phloem apoplasm.
4. However, the low PD frequencies between VP and CC or VP and ST does not rule out the possibility of some symplasmic loading of ST.
5. Transverse veins can contain either STs or TSTs, each of which is capable of transport, which suggests that retrieval and balancing sucrose concentration in adjacent parallel veins is facilitated by transverse vein ST or TST.
6. The evolution of this dual phloem system may have lead to a more efficient long-distance transport system in monocotyledons, compared with the collateral vein system in the majority of dicots.

Finally, information about the TSTs remains limited. We still know little about their ontogeny or whether the cell walls are non-lignified or if these cell walls undergo lignification with age. The relationship between TSTs and adjoining STs, is as yet not fully understood, neither is the role of the TSTs in assimilate distribution and transport. More research is needed to uncover information about the neglected phloem in monocot leaves.

### Conflict of interest statement

The author declares that the research was conducted in the absence of any commercial or financial relationships that could be construed as a potential conflict of interest.

## Abbreviations

BS: bundle sheath
CC: companion cell
LSCM: laser scanning confocal microscopy
Me: mesophyll
MS: mestome sheath
MX: metaxylem vessel
PPU: pore-plasmodesma unit
PD: plasmodesmata
SE-CC (complex): sieve element-companion cell complex
ST-CC: thin-walled sieve tube—companion cell complex
ST: thin-walled sieve tube
TST: thick-walled sieve tube
VP: vascular parenchyma
XVP: xylem-associated vascular parenchyma.
